# Hemoglobin levels and anemia evaluation among pregnant women in the remote and rural high lands of mid-western Nepal: a hospital based study

**DOI:** 10.1186/s12884-020-02870-7

**Published:** 2020-03-23

**Authors:** Deepak Sharma, Kapil Amgain, Prem Prasad Panta, Bishal Pokhrel

**Affiliations:** 1 Department of Physiology and Biophysics, Karnali Academy of Health Sciences (KAHS), Jumla, Nepal; 2 Department of Anatomy, Karnali Academy of Health Sciences (KAHS), Jumla, Nepal; 3 School of Public Health, Karnali Academy of Health Sciences (KAHS), Jumla, Nepal

**Keywords:** Highlands, Hemoglobin concentration, Pregnant women, Anemia

## Abstract

**Background:**

Anemia though is a major risk factor for unfavorable pregnancy outcomes; no previous studies have yet described the hemoglobin (Hb) concentrations and anemia prevalence among pregnant women of remote mid western highlands of Nepal where the aggravating factors that increase the risk of anemia are very common. In addition, the physiological adaptive Hb rise to altitude was considered in the study while evaluating anemia. Thus, our primary objectives were to study the hemoglobin levels and prevalence of anemia among pregnant women of Jumla and its adjoining districts, and to assess the potential associations of hemoglobin and anemia with women’s characteristics.

**Methods:**

The study was conducted in 319 singleton term non-smoker pregnant women who visited to the teaching hospital for delivery. Their blood samples were tested for Hb and related sociodemographic information was collected. One-way analysis of variance (ANOVA) and independent t-test were used to compare the mean Hb levels. Multiple linear regression model and multiple logistic regression model were used to assess the association of Hb level and anemia with pregnant women’s characteristics. The prevalence of anemia was calculated based on the altitude and pregnancy-adjusted Hb cut off value for anemia [{11+ adjustment factor (1.3)} gm./dl].

**Results:**

The overall mean hemoglobin concentration was (13.497 ± 1.64) gm/dl, ranging from 8 to 19.20 g/dl. The pregnant women Hb level showed significant association with their age (Coeff = 0.059; 95% CI: 0.011, 0.106; *p* = 0.015) and parity (Coeff = − 0.21; 95% CI: − 0.382, − 0.038; *p* = 0.017). The overall prevalence of anemia in the study population was 17.9% (57/319), which varied with age, parity and ethnicity. The *disadvantaged Janajati*s were more likely (OR = 4.615, 95% CI: 1.48, 14.35, *p* = 0.008) to have anemia compared to upper cast group.

**Conclusion:**

The mean Hb concentration was high and prevalence rate of anemia was low among pregnant women in karnali zone compared to average Nepali pregnant women. Women’s age and parity were significant predictors of Hb level. Ethnicity, however, was associated with the occurrence of anemia.

## Background

Anemia, a critical health problem during pregnancy, affects developing as well as developed countries [1]. The prevalence of anemia is 30.2% in non-pregnant women and 41.8% in pregnant woman around the world, with major share from the developing countries. Around, more than half of pregnant women in developing countries suffer from anemia [2]. This has become the major risk factor for adverse pregnancy outcomes; premature labor, low birth weight, maternal mortality and perinatal mortality [3]. Anemia, as defined by the world health organization (WHO), is a condition in which the number of red blood cells or their oxygen-carrying capacity is insufficient to meet the physiologic needs [4]. The hemoglobin (Hb) concentration of less than 11 g/dl, among pregnant women residing at an altitude of 1000 m or below, is considered as anemia [5]. Poor or insufficient diet, low socio-economic status [6], inadequate health services, illiteracy [7], deficiency of essential elements (like, iron, vitamin A, vitamin B12 and folic acid), recurrent pregnancies etc. [8] are the identified aggravating factors that increase the risk of anemia.

Normal hemoglobin concentration varies with age, sex, life style, race/ethnic, socioeconomic status, nutritional status, altitude, smoking and regional difference [4, 9, 10]. Ethnic groups due to their own unique habits, geographic conditions, and human genetics, may have major effects on Hb concentrations. The literatures have shown the racial differences of Hb distribution between African Americans and Whites [11, 12]. The altitude has remarkable effect on Hb concentration; with increase in height, environmental oxygen partial pressure gradually declines leading to lower oxygen saturation of the blood, and as an physiological adaptive response, hemoglobin level rises to meet the oxygen demand of the body. Thus, understanding the relationship between the Hb concentration and altitude is crucial for precisely estimating the occurrence of anemia in high altitude dwellers [1, 3].

Malnutrition has been a serious problem in Nepal and is a major threat particularly to the health of adolescent girls, pregnant and lactating mothers, 18% of the total women in Nepal are malnourished as indicated by the Nepal Demographic and Health Survey report (NDHS 2011). The nutritional status of pregnant women is seemingly very poor, predominantly in the remote hilly areas of Nepal, one of them being the Karnali zone [13]. The Karnali zone (mid - western Nepal), most backward and remote region of the country, comprises five high mountainous districts (above 2500 m) of karnali province, and is the home of different ethnic groups. The people are living in conditions of abject poverty, food crisis (the region is hit hard by drought and snowfall for months every year), and malnutrition along with other predisposing factors, like inaccessibility to health facilities, and recurrent pregnancies, all of which increase the possibility of occurrence of anemia [13, 14]. Far from nutritious food, the pregnant women as well as lactating mothers do not even get enough food; as a result, they face problems while giving birth to babies [14]. The several studies around the world also have reported the significant relationship between pregnancy Hb level and neonatal birth weight [15, 16].

Despite the above facts, to our knowledge there have been no previous studies assessing hemoglobin concentration, and evaluating prevalence of anemia among pregnant women of Karnali zone, the country’s most cheated place both by geography and development. Thus, our primary objectives were to study the hemoglobin levels and prevalence of anemia among pregnant women of the zone, and to evaluate the effects of women’s age, parity, and ethnicity on hemoglobin level and on the occurrence of anemia. Our secondary objective was to assess the relationship between maternal term hemoglobin concentration and neonatal birth weight. The outcomes of the study would alarm the concerned authorities for proper interventions in the region.

## Methods

### Setting

The study was conducted in the teaching hospital of Karnali Academy of health sciences (KAHS), Jumla (2514 m above the sea level), Karnali. The Karnali, one of seven federal provinces, divided into ten districts, occupy the higher mountains land of north and mid-hills of Nepal, and has been confronting the extreme difficulties of the remoteness [17, 18]. As per the Nepal Human Development Report 2014, Karnali’s Human Development Index (HDI) value increased from 0.347 to 0.445 between 2001 and 2011, but it was still the lowest compared to other parts of the country [19]. The KAHS teaching hospital, the only tertiary hospital in the province, provides antenatal, maternal and newborn health services largely to the people of Jumla and its four (Humla, Dolpa, Kalikot, Mugu) adjoining districts [20]. These five districts, earlier together called karnali zone, extends from 2500 to 4000 m in elevation, and have the population of 4,26,026 [21].

### Sample size

As an exploratory investigation, this hospital based cross sectional study was performed on 319 normal singleton non-smoker term pregnant women who visited to KAHS-teaching hospital for delivery from April to September 2018. The prevalence rate of anemia among pregnant women residing in the high hills of Nepal (15.6%) was considered for sample size estimation [22]. After reviewing the past records of the gynecology and obstetrics department, a six-month period was taken to include 316 participants, as calculated.

### Participants and measurements

The participants were from the five remotest precipitous mountainous districts of Karnali state - Jumla, Humla, Dolpa, Kalikot and Mugu. The mean age of the participants was (23.18 ± 4.87) years, ranging from 16 to 50 years. Almost half of the participants (49.2%) were nulliparous, the mean parity was 0.93 ± 1.35, ranging from 0 to 10. Due to remoteness of the region, women even have to walk for 72 h from the nearby districts to reach the teaching hospital. So, many of them were reluctant to visit hospital during their first and second trimesters.

Capillary blood, obtained by pricking the middle or ring finger of the non-dominant hand of each participant, was used for the Hb estimation. The puncture was made slightly off center - near the side, where the skin is thinner, with fewer nerve endings and less pain sensation. The first and second drops of blood were wiped away, as these drops show high degree of Hb variability, independent of the analytical device used for the test. The third drop was taken and Hb concentration was estimated by Hb photometer (B-Hemoglobin, precision of 1 g/L, HemoCue AB, Sweden). In this study, anemia was defined as a Hb level lower than the “Normal” (<11.0 g/dl) Hb level at the defined altitude [4]. Above 1000 m height, hemoglobin concentration is known to increase to compensate for the lowered partial pressure of oxygen and reduced oxygen saturation of blood. Based on the UNICEF, UNU, WHO documents 2001, to assess the prevalence of anemia in pregnant women, the adjustment factor 1.3 was added to the anemia cutoff value, in order to obtain the comparative cut off value at altitude 2250–3000 m [5]. Thus, the anemia cut off value for pregnant women in this study was 12.3 g/dl.

A questionnaire was used to collect each participant’s characteristics those were of greatest interest to us, i.e., name, age, address, parity, and ethnicity. The participants were then classified by ethnicity, age and parity. Ethnicity was divided according the government of Nepal and details were as follows: *Dalits (hills of Kami, Damai, Sharki, Gaine, Badi), Disadvantaged Janajati (hills of Magar, Tamang, Rai, Limbu, Sherpa, Bhote, Walung, Sunuwar, Kumal, Jirel, Danuwar, Thami, Raji), and Upper cast groups (Brahmin, Chhetri, Thakuri, Sanyashi, Raajput, Kayastha, Baniya, Marwadi, Jaire, Nurang, Bengali)* [18]. After delivery, the neonatal birth weight was recorded following standard protocol.

This study was conducted according to the guidelines of the declaration of helsinki and approved by the ethical committee of the institute. Pregnant women who agreed to participate in the study signed the consent form.

### Data analysis

The distribution of data was confirmed by Kolmogorov-Smirnov test before using parametric tests. One-way analysis of variance (ANOVA) was used to compare the mean hemoglobin levels among different ethnic groups, and parity. The difference of Hb levels between the age groups was tested by the independent t-test. Simple and multiple linear regression models were used to assess the influence of women’s characteristics (age, ethnicity and parity) on Hb level. Maternal age and parity were analyzed as continuous variable in this model.

Likewise, multiple logistic regression model was used to analyze the association of women’s characteristics with the occurrence of anemia. The relationship between maternal hemoglobin concentration and neonatal birth weight was derived from Pearson correlation analysis, and the equation was obtained from regression analysis.

Data were analyzed by SPSS version 20 (Statistical Package for Social Science, Inc., Chicago, USA). All statistical tests were two-tailed with a significance level of 0.05.

## Results

### Hemoglobin level and its association with women characteristics

**Fig. 1:**
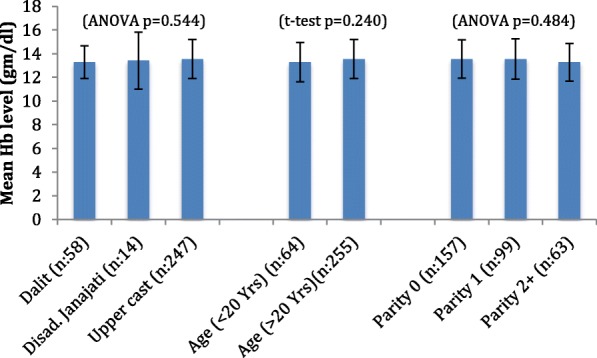
Hb levels and women characteristics

The overall mean hemoglobin concentration (*n* = 319) was (13.497 ± 1.64) gm/dl, ranging from 8 to 19.20 g/dl. The study showed no significant ethnicity, age and parity wise differences in Hb distributions in our study population (Fig. 1).

Regarding association between women’s characteristics and Hb level, the simple linear regression analysis showed that the characteristics (age, parity and ethnicity) independently were not the predictor of Hb level. However, the characteristics when taken together showed significant association with the Hb level. The results from multiple linear regression analysis of Hb are presented in Table 1. The table shows significant association of Hb level with age (Coeff = 0.058; 95% CI: 0.011, 0.106; *p* = 0.016) and parity (Coeff = − 0.21; 95% CI: − 0.382, − 0.037; *p* = 0.017). The women’s age and parity together was found to be the significant predictor of their Hb level.

**Table 1:** Correlates of hemoglobin level with women characteristics

Characteristics	Multiple linear regression analysis Coeff^a^ (95% CI)	*P* value
Ethnicity	0.090 (−0.143, 0.323)	0.449
Age of pregnant women	0.058 (0.011, 0.106)	0.016
Parity	- 0.21 (−0.382, − 0.037)	0.017

^a^*Coeff* regression coefficient, *CI* confidence interval

### Anemia and its association with women characteristics

Based on the altitude and pregnancy-adjusted anemia cut off value, the overall prevalence of anemia (*n* = 319) was 17.9% (*n* = 57). The prevalence differed by ethnicity, parity and age (Table 2). The *Disadvantaged Janajati* had the highest prevalence (42.9%) followed by *Dalits* (22.4%) and Upper cast group (15.4%). The women of lower age had greater prevalence (23.4%) than that of higher age (16.5%). Considering parity, the prevalence was highest in multiparous women (22.2%).

**Table 2:** Distribution of anemia (after altitude adjustment) by characteristics of women and odds ratios of anemia from multiple logistic regression model analysis (*N* = 319)

Characteristics (n)	Prevalence of anemia (n) (%)	Multiple logistic regression analysis odds ratio (95% CI)	*P*-value
Ethnicity			
Dalit (58)	13 (22.4%)	1.47 (0.72, 3.02)	0.288
Disadvantaged Janajati (14)	6 (42.9%)	4.615 (1.48, 14.35)	0.008
Upper cast group (247)	38 (15.4%)	–	
Age			
< 20 Yrs (64)	15 (23.4%)	1.77 (0.84, 3.72)	0.129
> 20 Yrs (255)	42 (16.5%)	–	
Parity			
0 (157)	29 (18.5%)	0.674 (0.30, 1.48)	0.328
1 (99)	14 (14.1%)	0.526 (0.22, 1.22)	0.138
2+ (63)	14 (22.2%)	–	
Total	57 (17.9%)		

The results from multiple logistic regression are presented in Table 2. The table shows that *Disadvantaged Janajatis* were more likely (OR = 4.615, 95% CI: 1.48, 14.35, *p* = 0.008) to have anemia than the Upper cast group of the region. The Dalits of the region were not more prone (OR = 1.47, 95% CI: 0.72, 3.02, *P* = 0.288) to anemia compared to the same group. Age and parity were not significantly associated with the occurrence of anemia.

Hosmer - Lemeshow test was done to check the goodness of fit of the model (*p* = 0.808), and the power of test calculated from the same was 81.8%.

### Correlation between term pregnancy Hb level and neonatal birth weight

**Fig. 2:**
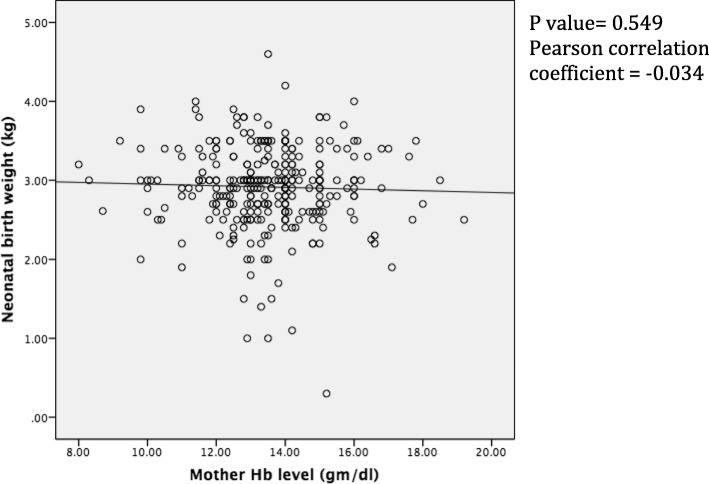
Graph depicting the dependence of birth weight on term pregnant women Hb concentration. The regression equation describing this relationship is: Effect on birth weight (g) = 3058.106 − [10.6 x term pregnant women Hb concentration (gm/dl)]

There was no association between the neonatal birth weight and the maternal term Hb level (r = − 0.034, *p* = 0.549) (Fig. 2).

## Discussion

In this study, the mean hemoglobin level among the pregnant women of the five mountainous (2500 – 4000 m) districts of mid-western Nepal was (13.49 ± 1.64) gm/dl, higher than the 12.1 g/dl among rural pregnant women living at 1300–2200 m altitude in Tanzania [23], and l2.76 g/dl among Tibetans of high lands [3]. The value, however, was slightly lower compared to the women of Bolivia (13.71 g/dl) dwelling at an altitude of 3600 m [24]. The inconsistency seen in the results can be attributed, but not limited, to geographical, racial, nutritional, and socio-economic variations. More participation of nulliparous, higher aged, and upper cast pregnant women in our study population, in supplement to physiological adaptive rise to decreased environmental oxygen, may have resulted into higher Hb level. The value obtained in our study was higher than that of WHO survey for mean Hb concentration of average Nepali pregnant women estimated at 11.1 g/dl (CI: 10.8–11.5 g/dl) [25].

This study showed no differences in Hb level by ethnicity, age and parity. The results contradict the findings of Zhang et al. who reported the ethnic differences of the Hb distribution in the 10 ethnic groups in China [26]. Likewise, the Hb level differences were reported in pregnant Tibetans (12.66 g/dl) and non-Tibetans (13.46 g/dl) [3]. Absence of ethnic differences in our study population may be due to the ethnic groups belonging to the same geographical conditions with alike nutritional status and feeding habits.

The women characteristics i.e. age, parity, and ethnicity independently were not associated with their Hb level. However, multiple linear regression analysis showed the pregnant women’s age and parity as significant predictor of the Hb level, whereas ethnicity had no significant association. The multiple regression equation describing this relationship between Hb level and women’s characteristics is:

Pregnant women Hb level (gm/dl) = 12.10 + 0.058. Age – 0.21.Parity + 0.090.Ethnicity (Ethnicity code: 1 = Dalits, 2 = disadvanated Janajati, 3 = upper cast group).

The overall prevalence of anemia in this study was 17.9% (*n* = 57), a mild public health problem according to the WHO classification of the public health importance of anemia [10]. The prevalence of anemia in pregnancy varies in women with different socio-economic conditions, lifestyles, ethnicity, parity or health-seeking behaviors across different cultures [27, 28]. Notably, the prevalence of anemia in our study population varied with ethnicity, age and parity. The *Disadvanted janajati*s had the highest prevalence followed by *Dalits* and Upper cast. Similarly, low aged, and multiparous women were more anemic.

Referring to the multiple logistic regression analysis, disadvantaged *Janajati* and *Dalits* pregnant women dwelling at high altitude areas of mid-western Nepal were more likely to have anemia than women of upper cast group, which was not an unexpected finding in our study setting. The western high remote mountainous region of Nepal still retains its centuries-old caste system; Dalits and Janajatis, the discriminated people under this system suffer from restriction on the use of public amenities, deprivation of economic opportunities, and generally neglected by the society. On top of this, illiteracy, malnutrition, inaccessibility to health facilities, and traditional restrictive beliefs widely existing among them may also have contributed to more anemia susceptibility [13].

To our knowledge, this is the first study that investigated the distribution of Hb levels and prevalence of anemia among pregnant women of high mountains (2500–4000 m), mid-western Nepal. Because of inaccessible scattered population in mountains, we were unable to perform a community-based study in the selected districts, thus this hospital based cross-sectional design, which involved imbalanced ethnic participants, and pregnant women all at their third trimester, may have underestimated the real prevalence of anemia, and may not provide direct epidemiological inference. However, the prevalence of anemia and its association to women characteristics shown by this study, though only reflects the tip of the iceberg, provides useful information and indicates a need for further anemia studies in these communities, and plan appropriate interventions to reduce the possibilities of unfavorable pregnancy outcomes. Disadvantaged ethnic groups, i.e. Dalits and janajatis, due to social discriminations and cultural restrictive customs barely seek hospital services, and this accounts for their less participation in the study. Our study sample involved 4.38% (*n* = 14) disadvantaged Janajati, 18.18% (*n* = 58) Dalits, and 77% (*n* = 247) upper cast participants. The proportion of these three sub-populations in Karnali province is 15, 23 and 62% respectively [29]. Furthermore, unobserved confounding factors in the hospital may have limited our understanding of the true prevalence of anemia.

Regarding the association between pregnancy Hb level and neonatal birth weight, this study showed the tendency of the inverse relation. As suggested by a correlation study, for every rise of hemoglobin concentration by 1.0 g/dl, the birth weight reduces by 0.038 kg [15]. This is justifiable with the well-known effect of high-altitude exposure during pregnancy that increases hematocrit and subsequently the blood viscosity, which lowers birth weight as a higher blood viscosity is a risk factor for sub optimal placenta-perfusion [16].

## Conclusion

The mean Hb concentration was higher, and anemia prevalence rate was low among pregnant women of high mountains, mid-western Nepal, compared to average Nepali pregnant women. Women’s age and parity were found to be the significant predictors of hemoglobin level. The prevalence of anemia varied with ethnicity, age, and parity. The occurrence of anemia was associated with the ethnicity in the study population. The disadvantaged Janajati women were more likely to have anemia compared to upper cast group. Our study indicates a need of the extensive community based study in remote mountains, in order to accurately determine the degree of anemia and its association with women characteristics.

## Data Availability

The datasets obtained and/or analyzed during the current study are not publicly available due to confidentiality consent of the study but can be obtained from the corresponding author on reasonable request.

## Abbreviations

CI: Confidence interval
Coeff: Regression coefficient
Hb: Hemoglobin
KAHS: Karnali Academy of Health Sciences
OR: Odds Ration
UNICEF: United Nations Children’s Fund
UNU: United Nation University
WHO: World Health Organization
